# Breakthrough Infections of SARS-CoV-2 Gamma Variant in Fully Vaccinated Gold Miners, French Guiana, 2021

**DOI:** 10.3201/eid2710.211427

**Published:** 2021-10

**Authors:** Nicolas Vignier, Vincent Bérot, Nathalie Bonnave, Sandrine Peugny, Mathilde Ballet, Estelle Jacoud, Céline Michaud, Mélanie Gaillet, Félix Djossou, Denis Blanchet, Anne Lavergne, Magalie Demar, Mathieu Nacher, Dominique Rousset, Loïc Epelboin

**Affiliations:** Institut Pierre Louis d’Épidémiologie et de Santé Publique Inserm UMR1136, Paris, France (N. Vignier);; Université Sorbonne Paris Nord, Bobigny, France (N. Vignier);; Centre Hospitalier de Cayenne, Cayenne, French Guiana (N. Vignier, V. Bérot, N. Bonnave, S. Peugny, E. Jacoud, C. Michaud, M. Gaillet, F. Djossou, D. Blanchet, M. Demar, M. Nacher, L. Epelboin);; Centre d’Investigation Clinique Antilles Guyane Inserm 1424, Cayenne (N. Vignier, M. Nacher, L. Epelboin);; Centre Hospitalier Ouest Guyanais, Saint Laurent du Maroni, French Guiana (V. Bérot);; Agence Régionale de la Santé de Guyane, Cayenne (M. Ballet);; Institut Pasteur de la Guyane, Cayenne (A. Lavergne, D. Rousset)

**Keywords:** COVID-19, SARS-CoV-2, severe acute respiratory syndrome coronavirus 2, coronavirus disease, viruses, respiratory infections, zoonoses, vaccines, variants, Gamma variant of concern, BNT162 vaccine, breakthrough, outbreak, gold miner, French Guiana

## Abstract

An outbreak of severe acute respiratory syndrome coronavirus 2 caused by the Gamma variant of concern infected 24/44 (55%) employees of a gold mine in French Guiana (87% symptomatic, no severe forms). The attack rate was 60% (15/25) among fully vaccinated miners and 75% (3/4) among unvaccinated miners without a history of infection.

On May 31, 2021, a gold miner tested positive for severe acute respiratory syndrome coronavirus 2 (SARS-CoV-2) at the Cacao health center, French Guiana. He worked in a legal gold mine located 72 km from Cayenne (including 13 km of forest trail) in the Amazon Forest. Other workers from the same site were reported as symptomatic, although a large part of this specific population had benefited from complete coronavirus disease (COVID-19) vaccination in the previous month. A medical team went on site to investigate, examine, and screen the 44 employees of the mine. We describe results of the outbreak investigation.

## The Study

We collected data by completing standardized forms with data gathered through interviews and medical examination of all gold miners and by reviewing the health center records. All employees of the mine were examined by a physician and screened by nasopharyngeal Panbio COVID-19 Ag Rapid Test device (Abbott Laboratories, https://www.abbott.com) if they were symptomatic; all miners underwent SARS-CoV-2 PCR EurobioPlex SARS-CoV-2 Multiplex (Eurobio Scientific, https://www.eurobio-scientific.com) testing on June 2, 2021. All employees were reassessed on June 8 and 15, 2021; those with negative results were rescreened by PCR. We performed serologic tests on blood specimens collected from 39 gold miners whether PCR was negative or positive. Serum samples were initially tested with anti–SARS-CoV-2 ELISA IgG (Euroimmun, https://www.euroimmun.com). We used descriptive statistics to analyze the variables and performed univariate analyses. Intervention was a public health response as part of activities of Cayenne Hospital. All gold miners gave their verbal informed consent for recording and processing of information during interviews and for the use of their biologic results for research purposes, and physicians completed a nonobjection form in accordance with laws of France.

Mine workers were mostly men (42/44); median age was 53.3 years. Eighteen of the workers had risk factors for severe COVID-19: high blood pressure (11/44), diabetes mellitus (4/44), or obesity (4/44). Miners lived onsite in separate rooms but shared face-to-face meals and machine cabins. They also worked outside without masks. Twenty-one workers reported contacts outside the mining site during the previous 2 weeks.

The first symptomatic cases occurred on May 29 among 3 machine operators and 1 miner. Their antigen tests and PCR results were positive on June 2. The peak of the outbreak occurred 2 days after the early cases, on May 31 (Figure). Five asymptomatic miners who tested negative moved to a separate open housing for quarantine. Among them, 4 became symptomatic during June 6–8 and tested positive on June 8. 

**Figure:**
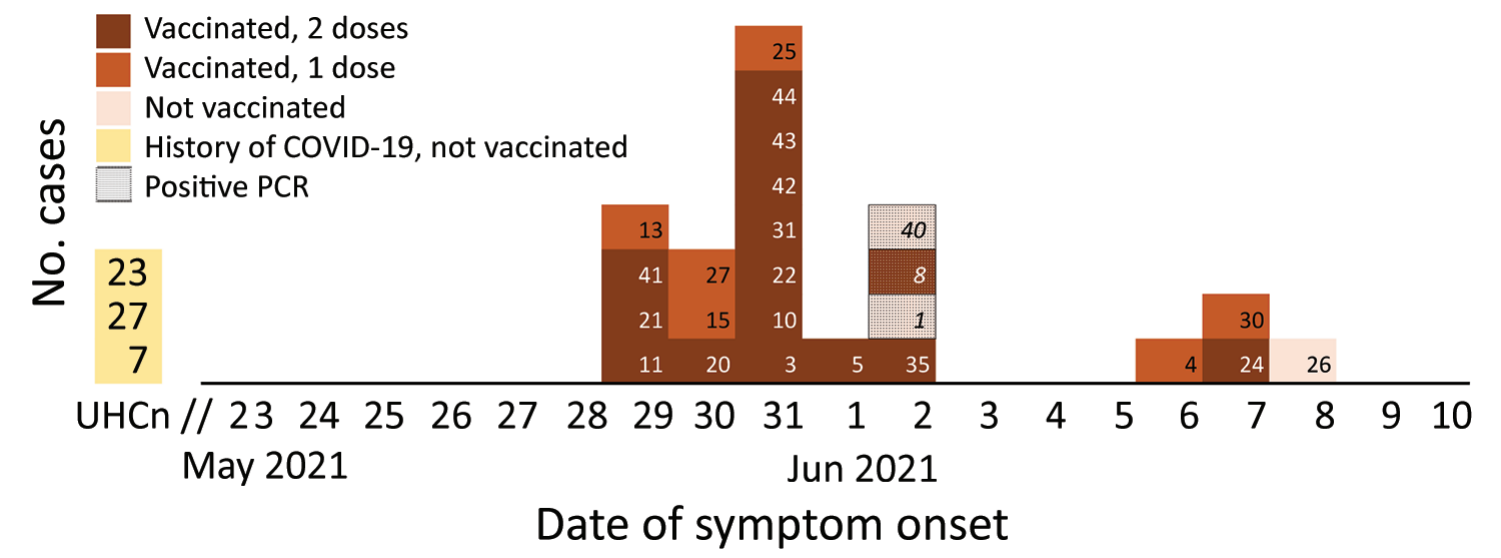
Epidemic curve for symptomatic and asymptomatic COVID-19 case-patients, by date of symptom onset or date of PCR, during an outbreak in gold-mine workers in French Guiana, May 29–June 8, 2021. Of the case-patients with undated history of COVID-19, case-patient 7 had a high level of severe acute respiratory syndrome coronavirus 2 (SARS-CoV-2) antibodies, probably from an old infection. Case-patients 23 and 26 had low levels of antibodies, indicating either recent or very old infection. Case-patient 40 was positive by PCR with cycle threshold = 33 on June 2 and had a high level of SARS-CoV-2 antibodies, indicating possible semi-recent infection dating back a few days or weeks. COVID-19, coronavirus disease; UHCn, undated history of COVID-19 (positive serology) with negative PCR, not vaccinated.

The overall attack rate was 54.5% (24/44); 87% were symptomatic, 65% with fever, and 22.6% with dyspnea. No clinically severe COVID-19 (*1*) was observed, and no patient was hospitalized. Among infected miners, 18/23 (78.2%) had a cycle threshold (C_t_) <28 (Appendix Table 1). The variant of concern (VOC) gamma (P.1 lineage), also known as 20J/501Y.V3, was identified in 9/9 viruses sequenced by the Pasteur Institute (Appendix Table 2), without any new mutation. Of the 4 persons who tested negative and were not vaccinated, 3 had a positive SARS-CoV-2 IgG. Patient 40 could be the index case-patient; he reported visiting his family the previous week and had an asymptomatic SARS-CoV-2 infection with C_t_ of 33–35.

Regarding immune status, 25/44 (56.8%) were fully vaccinated with BNT162b2 vaccine (Pfizer-BioNTech, https://www.pfizer.com); 3/6 workers who had a history of COVID-19 were vaccinated with a single injection, according to the guidelines of France (*2*) (Table). Several BNT162b2 batch numbers were involved. Vaccine temperature had been monitored and electronically recorded by LogTag Analyzer (LogTag Recorders, http://www.logtag-recorders.com) without any break in the cold chain. The attack rate was 15/25 (60.0%) in fully vaccinated miners, 6/15 (40.0%) in those partially vaccinated or with a history of COVID-19, and 3/4 (75%) in those not vaccinated. Attack rate was 0/6 among persons with a previous history of COVID-19 versus 63.2% among those with no previous history (Table). No other factors were found to be associated with the risk for infection. 

**Table:** Characteristics of gold miners by active SARS-CoV-2 infection status, Cacao, French Guiana, May–June 2021*

Characteristic	Total, no. (%)		Acute SARS-CoV-2 infection			
			No	Yes	Total %	p value
All participants	44		20	24	54.6	
Mean age	44		51.9	54.5		0.88
Immune status						
Fully vaccinated, 2 doses	25 (56.8)		10	15	60.0	0.20
Vaccinated, 1 dose	9 (20.5)		3	6	66.7	
History of COVID-19, vaccinated 1 dose	3 (6.8)		3	0	0.00	
History of COVID-19, not vaccinated	3 (6.8)		3	0	0.00	
Neither vaccinated nor history	4 (9.1)		1	3	75.0	
History of previous COVID-19						
Y	6 (8.9)		6	0	0.00	0.004
N	38 (86.4)		14	24	63.2	
Sex						
M	42 (95.4)		18	24	57.1	0,11
F	2 (4.5)		2	0	0.0	
Age, y						
<55	24 (54.5)		11	12	52.2	0.74
>55	20 (45.4)		9	12	57.1	
Country of birth						
Brazil	34 (77.3)		16	18	52.9	
Surinam	6 (13.6)		2	4	66.7	
Haiti	2 (4.5)		1	1		
Guyana	1 (2.3)		0	1		
France	1 (2.3)		1	0		
Occupation						
Laborer	20 (45.4)		8	12	60.0	0.73
Operator	17 (38.6)		8	9	52.9	
Other	7 (15.9)		4	3	42.9	
Eat alone						
Y	7 (15.9)		5	2	28.6	0.13
N	37 (84.1)		15	22	59.5	
Live alone						
Y	28 (63.6)		15	13	46.4	0.13
N	16 (36.4)		5	11	68.8	
Contact outside the mine in the previous 2 weeks						
Y	26 (60.5)		10	16	61.5	0.35
N	17 (39.5)		9	8	47.1	
Underlying conditions						
Hypertension	11 (25.0)		5	6	54.6	1.00
Diabetes	4 (9.1)		2	2	50.0	0.85
Obesity	4 (9.1)		2	2	50,0	0.85
Cardiac insufficiency	3 (8.3)		1	2	66.7	0.62

*We defined acute SARS-CoV-2 infection in participants as having a positive SARS-CoV-2 antigenic or PCR test in June 2021, symptomatic or not. Of the 24 with acute infection, 21 were symptomatic and 3 asymptomatic infections. p value indicates degree of significance. COVID-19, coronavirus disease; SARS-CoV-2, severe acute respiratory syndrome coronavirus 2.

Among the mine workers were recorded 14/28 vaccine clinical failures (COVID-19 onset >14 days after the second dose, or after a single dose for patients with history of COVID-19); none had serious infections. Twelve (42.3%) of the 28 fully vaccinated reported vaccine reactogenicity. Among the fully vaccinated, the SARS-CoV-2 IgG ratio was high for most (mean 9.22, SD 1.5). We performed serologic testing a median of 4 (interquartile range [IQR] 2–5.5) days after the onset of symptoms in symptomatic patients and 58 (IQR 46–62) days after vaccination.

mRNA vaccines such as BNT162b2 demonstrated high effectiveness both in clinical trials and in real-world situations against wild-type SARS-CoV-2 and its Alpha variant infections (*3*,*4*). However, other VOC, such as Beta or Gamma, harbor mutations conferring potential escape from humoral response induced either by prior infection or vaccination, as proven by both decreased seroneutralization in vitro (*5*–*7*) and in vivo by observational studies in the case of the Beta variant (*8*,*9*). However, such breakthrough infections, even those caused by Beta variant, remain rare in fully vaccinated populations and are mostly asymptomatic or moderately symptomatic (*8*–*10*).

## Conclusions

We describe a COVID-19 Gamma variant cluster with a high attack rate even in fully vaccinated persons. The Gamma variant is the predominant variant in French Guiana which, as of July 2021, caused a third epidemic wave, threatening to overwhelm the hospital capacity (*11*). Such a low vaccine efficiency against infection by the Gamma variant was not expected because in vitro studies have shown a similar reduction of neutralization for Beta or Gamma variants by BNT162b2-elicited antibodies (*5*) and a conserved CD4+ T-cell response against spike proteins from the Beta variant (*6*). Of the 10,262 COVID-19 vaccine breakthrough infections identified in the United States during January–April 2021, for which 555 had available sequencing, only 28 were caused by the Gamma variant (*12*). Furthermore, real-world effectiveness against any infection by a Beta variant, which shares a similar E484K mutation on the gene coding for the spike protein, was estimated at 75.0% (*9*). Given the surprisingly high attack rate, we hypothesized potential dysfunctions of conservation or administration of vaccines, but the absence of traceable cold-chain interruption and the use of different batches seemed to refute this hypothesis. The relative isolation of the mining site and careful contact tracing suggested limited numbers of viral introductions inside this community. The low C_t_ of positive PCR for SARS-CoV-2 despite prior vaccination suggested that a complete vaccination scheme with BNT162b2 vaccine was not sufficient to prevent symptomatic SARS-CoV-2 infection and its transmission in this context of communal life without masks. The working conditions of some miners (heat, humidity, aerosol) and the sharing of machine cabs for others could also have contributed to transmission. The absence of severe COVID-19 in a high-risk population (*13*) suggests but does not prove protection against severe disease, as reported for the Beta variant in another context (*14*).

In conclusion, we describe a VOC Gamma COVID-19 outbreak with a strikingly high attack rate among persons fully vaccinated with BNT162b2 vaccine. Our observation suggested that BNT162b2 protected from severe COVID-19. However, this single unexpected outbreak in a small and isolated vaccinated population requires further real-life studies on BNT162b2 vaccine effectiveness against the VOC Gamma. Masking and social distancing, even among those fully vaccinated, may be necessary among persons with frequent exposure in Gamma variant–endemic zones.

Appendix Additional information and sequencing results from breakthrough infections of SARS-CoV-2 Gamma variant in fully vaccinated gold miners, French Guiana.
