# Supplementary material for: Breakthrough Infections of SARS-CoV-2 Gamma Variant in Fully Vaccinated Gold Miners, French Guiana, 2021
Source: Emerg Infect Dis. 2021 Oct;27(10):2673–6. doi: 10.3201/eid2710.211427 (PMC8462339; doi:10.3201/eid2710.211427)
Supplement: Appendix — Additional information and sequencing results from breakthrough infections of SARS-CoV-2 Gamma variant in fully vaccinated gold miners, French Guiana. [file 21-1427-Techapp-s1.pdf]

Article DOI: https://doi.org/10.3201/eid2710.211427

Breakthrough Infections of SARS-CoV-2 Gamma Variant in Fully Vaccinated Gold Miners, French Guiana, 2021

Appendix

Nine confirmed samples of SARS-CoV-2 were submitted to whole-genome sequencing. Viral RNA was extracted from 150µL of nasal swabs using the QIAamp viral RNA Mini Kit (QIAGEN, https://www.qiagen.com) following the manufacturer's instructions. Extracted RNAs were subjected to whole-genome sequencing following the ARTIC network protocol version 3 (https://www.protocols.io/view/ncov-2019-sequencing-protocol-v3-locost-bh42j8ye).

In brief, the LunaScript RT SuperMix Kit (New England Biolabs, https://www.neb.com) was used to obtain single-strand cDNA for each sample. All cDNAs were amplified using the ARTIC network SARS-CoV-2 protocol with V3 primers. Prior to library preparation, the multiplex primer sets (divided into 2 separate pools) were mixed. Sequencing was carried out on the Oxford Nanopore MinION devices (DNA Links, https://www.dnalinkseqlab.com; Oxford Nanopore Technologies, https://nanoporetech.com), utilizing FLO-MIN106 flow cells and the SQL-LSK109 ligation sequencing kit (Oxford Nanopore Technologies). On the MinION device, barcoding was carried out with the EXP-NBD104 barcoding kit (Oxford Nanopore Technologies). Nanopore sequencing reads were basecalled with Guppy 3.4.5+fb1fbfb (Oxford Nanopore Technologies) and analyzed with the Nanopolish workflow provided by Artic (https://github.com/artic-network/artic-ncov2019.git, commit ddfb2dc87a4f442f821787ef90a92625f6bd6a09).

The sequences have been deposited on the GISAID website (https://www.gisaid.org).

Appendix Table 1. Data from analysis of coronavirus disease outbreak in a legal gold mine, French Guiana, June 2021*

No.	Date	Result	PCR technique	C_t (RdRp1)	C_t (RdRp2)	C_t (*N* gene)	Immune status	Symptomatic	Sequencing
1	2021 Jun 2	PCR positive	Eurobio	29	28	34	Non-immune	0	Not done
2	2021 Jun 2	0					Vaccinated 1 dose	0	
3	2021 Jun 2	PCR positive	Qiastat†	NA	NA	26,1	Vaccinated 2 doses	1	Gamma
4	2021 Jun 2	PCR positive	Eurobio	19	20	25	Vaccinated 1 dose	1	Gamma
5	2021 Jun 2	PCR positive	Eurobio	22	21	27	Vaccinated 2 doses	1	Gamma
6	2021 Jun 2	0					Vaccinated 2 doses	1	
7	2021 Jun 2	0					History of COVID-19	0	
8	2021 Jun 2	PCR positive	Eurobio	21	20	26	Vaccinated 2 doses	0	Gamma
9	2021 Jun 2	0					History of COVID-19	1	
10	2021 Jun 2	PCR positive	Eurobio	18	18	22	Vaccinated 2 doses	1	Gamma
11	2021 Jun 2	PCR positive		25	24	29	Vaccinated 2 doses	1	Gamma
12	2021 Jun 2	0					History of COVID-19 – Vaccinated 1 dose	1	
13	2021 Jun 2	PCR positive	Eurobio	25	25	30	Vaccinated 1 dose	1	Not done
14	2021 Jun 2	0					Vaccinated 2 doses	0	
15	2021 Jun 2	PCR positive	Eurobio	18	18	23	Vaccinated 1 dose	1	Not done
16	2021 Jun 2	0					Vaccinated 1 dose	0	
17	2021 Jun 2	0					Non-immune	0	
18	2021 Jun 2	0					Vaccinated 2 doses	1	
19	2021 Jun 2	0					Vaccinated 2 doses	0	
20	2021 Jun 2	PCR positive	Eurobio	28	27	32	Vaccinated 2 doses	1	Not done
21	2021 Jun 2	positive antigen test	NA	NA	NA	NA	Vaccinated 2 doses	1	NA
22	2021 Jun 2	PCR positive	Eurobio	19	19	24	Vaccinated 2 doses	1	Gamma
23	2021 Jun 2	0					History of COVID-19	0	
24	2021 Jun 2	PCR positive	Eurobio	20	21	26	Vaccinated 2 doses	1	Gamma
25	2021 Jun 2	PCR positive	Eurobio	24	23	28	Vaccinated 1 dose	1	Gamma
26	2021 Jun 2	PCR positive	Eurobio	25	25	30	Non-immune	1	Not done
27	2021 Jun 2	PCR positive	Eurobio	>36	NA	NA	Vaccinated 1 dose	1	Not done
28	2021 Jun 2	0					Vaccinated 2 doses	0	
29	2021 Jun 2	0					Vaccinated 2 doses	0	
30	2021 Jun 2	PCR positive	Eurobio	18	20	26	Vaccinated 1 dose	1	Not done
31	2021 Jun 2	PCR positive	Eurobio	23	23	27	Vaccinated 2 doses	1	Not done
32	2021 Jun 2	0					Vaccinated 1 dose	0	

No.	Date	Result	PCR technique	C_t (RdRp1)	C_t (RdRp2)	C_t (*N* gene)	Immune status	Symptomatic	Sequencing
33	2021 Jun 2	0					Vaccinated 2 doses	0	
34	2021 Jun 2	0					Vaccinated 2 doses	0	
35	2021 Jun 2	PCR positive	Eurobio	21	21	26	Vaccinated 2 doses	1	Not done
36	2021 Jun 2	0					Vaccinated 1 dose	0	
37	2021 Jun 2	0					Vaccinated 2 doses	1	
38	2021 Jun 2	0					Vaccinated 2 doses	0	
39	2021 Jun 2	0					Vaccinated 1 dose	0	
40	2021 Jun 2	PCR positive	Eurobio	35	33	No signal	Non-immune	0	Not done
41	2021 Jun 2	PCR positive	Eurobio	31	30	36	Vaccinated 2 doses	1	Not done
42	2021 Jun 2	PCR positive	Eurobio	25	25	29	Vaccinated 2 doses	1	Not done
43	2021 Jun 2	PCR positive	Eurobio	30	30	35	Vaccinated 2 doses	1	Not done
44	2021 Jun 4	PCR positive	Eurobio	27	26	31	Vaccinated 2 doses	1	Not done

*CT values were not different according to the immune status: median (interquartile range [IQR] of C_t (RdRp1) = 29 (25-35) for non-immune (n = 3), 21.5 (18-25) for vaccinated 1 dose (n=6) and 23 [21-27] for vaccinated 2 doses (n = 13), p = 0.15 with median test. For RdRp1: median [IQR] = 28 [25-33] for non-immune (n = 3), 20 [20-23] for vaccinated 1 dose (n = 5) and 23 (21-26) for vaccinated 2 doses (n = 13), p = 0.09 with median test. For N gene: median (IQR) = 32 (30-34) for non-immune (n = 2), 26 (25-28) for vaccinated 1 dose (n = 5), and 27 (26-31) for vaccinated 2 doses (n = 14), p = 0.30 with median test.
†PCR was performed with QIAstat-Dx respiratory panel for this patient. Only SARS-CoV-2 was detected.

Appendix Table 2. Sequencing data for 9 SARS-CoV-2 viruses from an outbreak in workers in a legal gold mine, Cacao, French Guiana, June 2021

GISAID accession ID	Collection date	Location	Host	Sex	Age	Last vaccinated	Passage	Specimen	Lineage	Clade	No.
EPI_ISL_2627852	2021 Jun 2	South America / French Guiana / Cacao	Human	M	53	2 doses Pfizer	Original	Nasopharyngeal swab	P.1	GR	3
EPI_ISL_2627854	2021 Jun 2	South America / French Guiana / Cacao	Human	M	42	1st dose Pfizer 2021 May 5	Original	Nasopharyngeal swab	P.1	GR	25
EPI_ISL_2627855	2021 Jun 2	South America / French Guiana / Cacao	Human	M	63	2nd dose Pfizer 2021 Apr 7	Original	Nasopharyngeal swab	P.1	GR	10
EPI_ISL_2627856	2021 Jun 2	South America / French Guiana / Cacao	Human	M	58	2nd dose Pfizer 2021 May	Original	Nasopharyngeal swab	P.1	GR	5
EPI_ISL_2627857	2021 Jun 2	South America / French Guiana / Cacao	Human	M	59	2nd dose Pfizer 2021 Apr 7	Original	Nasopharyngeal swab	P.1	GR	8
EPI_ISL_2627858	2021 Jun 2	South America / French Guiana / Cacao	Human	M	53	2nd dose Pfizer 2021 Apr 7	Original	Nasopharyngeal swab	P.1	GR	11

GISAID accession ID	Collection date	Location	Host	Sex	Age	Last vaccinated	Passage	Specimen	Lineage	Clade	No.
EPI_ISL_2627859	2021 Jun 2	South America / French Guiana / Cacao	Human	M	51	2nd dose Pfizer 2021 Apr 7	Original	Nasopharyngeal swab	P.1	GR	22
EPI_ISL_2629233	2021 Jun 8	South America / French Guiana / Cacao	Human	M	51		Original	Nasopharyngeal swab	P.1	GR	24
EPI_ISL_2629234	2021 Jun 8	South America / French Guiana / Cacao	Human	M	49		Original	Nasopharyngeal swab	P.1	GR	4
